# Medical University !

**Published:** 1846-02-01

**Authors:** 


*Medical University!*—Truly the wonders of steam in this enlightened age exceed all that could, by any effort of the imagination, have been anticipated! The Legislature of the State of Alabama have incorporated a *Medical University!* With our old fashioned notions we had supposed that this conjunction of terms involved a solecism, but we had forgotten the expansive powers of steam. The, so styled, Medical University, is a steam institution of the "purely Thomsonian order." The teachers say, that "an epoch in the history of Alabama will *bare* on the first Monday in Nov. 1845"—the lectures commencing at that time. The epoch is now passed some three months. It is time to "clear the track," and "look out for the engine!" There is something interesting in seeing a superb Humbug flourish. Success to the Steam Medical University of Alabama! *Vive la Bagatelle!*

